# PROFILE OF PATIENTS RECEIVING TOTAL KNEE ARTHROPLASTY: A CROSS-SECTIONAL STUDY

**DOI:** 10.1590/1413-785220172505168806

**Published:** 2017

**Authors:** MARCELO JOSÉ CORTEZ BEZERRA, IGOR MAGALHÃES BARBOSA, THALES GONÇALVES DE SOUSA, LARISSA MEIRELES FERNANDES, DIEGO LEONARDO MENEZES MAIA, LUCAS MEIRELES HOLANDA

**Affiliations:** 1. Department of Orthopedics and Traumatology, Universidade de Fortaleza (Unifor), Fortaleza, CE, Brazil.; 2. Centro Universitário Christus (Unichristus), Fortaleza, CE, Brazil.

**Keywords:** Arthroplasty, Knee, Osteoarthritis

## Abstract

**Objective:**

To describe the epidemiological profile, presented deformities, associated comorbidities, and impact on quality of life in patients with knee osteoarthritis. This study was conducted in a philanthropic hospital in Fortaleza from 2014 to 2015.

**Methods:**

Data were collected from medical records, epidemiological forms, and by applying the Lequesne index questionnaire, which contains several questions related to pain, discomfort and functional limitation to assess the severity of symptoms.

**Results:**

Females were more prevalent (76.7%), as were patients over 65 years of age (61.6%) and non-whites (81.6%). As for comorbidities, 83.3% had hypertension and 31.7% had diabetes. Of the total, 76.5% cases were genu varum, and 23.5% genu valgum. According to the Lequesne index findings, 61.6% cases were “extremely severe,” and women had higher scores.

**Conclusion:**

Females were more prevalent and whites were less prevalent. The most frequent comorbidity was hypertension. Female and elderly patients have more severe disease according to Lequesne index score, and these findings were statistically significant. ***Level of Evidence II, Prospective Study.***

## RESUMO

**Objetivo:**

Descrever o perfil epidemiológico, as deformidades, comorbidades associadas e o impacto sobre a qualidade de vida de pacientes com osteoartrite de joelho. O estudo foi realizado em um hospital filantrópico de Fortaleza, no período de 2014 a 2015.

**Métodos:**

A coleta de dados foi realizada a partir de análises de prontuários, uso de formulários epidemiológicos e aplicação do questionário de Lequesne, que tem várias questões sobre dor, desconforto e limitação funcional, para avaliar a gravidade dos sintomas.

**Resultados:**

Houve maior prevalência do sexo feminino (76,7%), de pacientes com mais de 65 anos (61,6%) e das raças não brancas (81,6%). Sobre comorbidades, 83,3% tinham hipertensão arterial, 31,7% tinham diabetes. Do total, 76,5% tinham geno varo e 23,5%, geno valgo. De acordo com o questionário de Lequesne, 61,6% tinham quadro “extremamente grave”, sendo as mulheres as que tiveram maior pontuação.

**Conclusão:**

O sexo feminino foi o mais prevalente. A raça branca foi a menos prevalente. A comorbidade com maior incidência foi a hipertensão arterial. O sexo feminino e os pacientes mais idosos apresentam maior gravidade da doença, segundo o questionário de Lequesne. Esses achados foram estatisticamente significantes. **Nível de Evidência II, Estudo Prospectivo.**

## INTRODUCTION

The growing number of procedures such as arthroplasty results from a number of factors such as the aging of the population, the increasing prevalence of rheumatoid arthritis, and increased numbers of obese patients.^1,2^

Total knee arthroplasty (TKA) is considered to be among the most successful types of orthopedic surgery, since even after 15 years implant survival exceeds 95%; furthermore, the improvement in quality of life is very significant.^3-5^

The main cause in most patients who undergo this procedure is osteoarthrosis.^6^ ^,^ ^7^

Studies suggest that Brazil will have the fifth-largest population on the planet in 2050,^8^ indicating that the frequency of TKA may increase over the next 30 years.

In order to understand the patients who undergo TKA to treat osteoarthritis (OA), this study collected Lequesne scores and a variety of data including epidemiological information from all patients with OA who were recommended for surgical treatment at the outpatient orthopedics clinic at Hospital Santa Casa de Misericórdia de Fortaleza. 

## MATERIALS AND METHODS

This transversal, descriptive study is based on quantitative data. It was carried out between January 2014 and January 2015 at the Santa Casa de Misericórdia de Fortaleza charity hospital. We included patients with osteoarthritis who were referred for surgical treatment and signed the informed consent form. Exclusion criteria were lack of data in the medical records and non-agreement to sign the informed consent form. The study was approved by the institutional review board under number CAAE 44595315.1.0000.5049.

 Initially data such as sex, race, origin, diagnosis, and presenting deformity were collected from the patient medical records for analysis. In addition, patients filled out an epidemiological form developed by the researchers (Annex 1) which collected data such as age, profession, smoking and drinking habits, wait time prior to surgery, and associated comorbidities such as hypertension and diabetes mellitus. Next, the Lequesne index questionnaire was applied prior to surgery. This questionnaire assesses pain and functional limitation and classifies patients by score. The severity of the disease in the patient can be classified as follows: “mild” (1-4 points), “moderate” (5-7 points), “severe” (8-10 points), “very severe” (11-13 points) and “extremely severe” (≥14 points). (Annex 2)

For uniform application of the questionnaires, the researchers were trained prior to administration.

The patients were recommended for surgical treatment after physical examination and imaging exams performed by orthopedic physicians and radiologists. At no time during this study did these physicians know the Lequesne index scores for their patients.

The statistical analysis was performed using the IBM SPSS Statistics software, version 20.0.0. A 5% significance level was adopted, and the chi-square hypothesis test was used to investigate the association between categorical variables and the distribution of the sample. P-values <0.05 were considered statistically significant.

## RESULTS

The initial sample consisted of 152 patients; at the end of the study, the sample was reduced to 60 patients with knee OA because of missing information in the medical records and loss to follow-up.

Women comprised 76.6% of the sample and men 23.3% (Table 1). Women were seen to be more affected according to Lequesne index score (P=0.034). Patients aged over 65 years were 61.6% of the sample, and 38.3% were in the 55-65 age range. Application of Pearson’s correlation coefficient showed that this correlation was significant (P=0.035) and the relationship was positive, demonstrating that increased age means higher index scores, and consequently the patient is more seriously affected (Table 2).

**Table 1:** Severity according to sex.

	Extremely severe	Very severe	Severe	Moderate
Total sample	61.6%	30%	6.6%	1.6%
Females P=0.034	53.3%	20%	1.6%	1.6%
Males	8.3%	10%	5%	0%

**Table 2:** Severity according to age.

P=0.035	Extremely severe	Very severe	Severe	Moderate
55-65 years	23.3%	11.66%	3.3%	0%
>65 years	38.33%	18.33%	3.3%	1.6%

As for comorbidities, 83.3% of the patients had hypertension, 31.7% had diabetes, and 26.7% had hypertension and diabetes. Of all the patients with a Lequesne score >14 (“extremely severe”), 78.3% had hypertension and 21.6% did not (Table 3); 8.3% had knee OA secondary to rheumatoid arthritis. With regard to alcohol and tobacco use, 20% of the patients drank alcohol and 16.7% were smokers. No significant connection was found between these habits and disease severity as measured by the Lequesne scores.

**Table 3:** Comorbidities and disease severity.

	Hypertension	Diabetes	Hypertension and diabetes
Total sample	84.9%	31.7%	26.7%
Extremely severe	48.3%	23.3%	18.3%
Very severe	28.3%	6.7%	6.7%
Severe	6.7%	1.7%	1.7%
Moderate	1.6%	0%	0%

Of the total, 76.5% of cases were genu varum and 23.5% genu valgum. According to the Lequesne index, 61.6% of the cases were classified as “extremely severe,” 30% as “very severe,” 6.6% as “severe,” and 1.6% (only one patient) as “moderate.” All patients who had OA secondary to rheumatoid arthritis had very high scores, such as 21 points. The highest patient score was 23 points, the lowest score was 7, and the average was 15.53. We also obtained information about wait time for each patient prior to surgery, which ranged from <1 year for 15% of patients and 1-5 years for 81.6% of patients to >5 years for 3.3% of patients.

## DISCUSSION

Many Brazilian studies involving patients who received TKA have found a high prevalence of female patients with an average age ranging from 69 to 79 years.^6^ ^,^ ^9^ ^,^ ^10^ As for race, the studies in Brazil are limited. International studies have found that non-whites (namely mixed-race people of African descent and Blacks) have more functional limitation due to OA, and non-white women are two times more likely to have knee OA.^11^ ^-^ ^13^ The present study found a greater prevalence of females (76.7%), patients over age 65 (61.6%), and non-white patients (81.6%), which is in line with most international studies. However, the Lequesne scores did not show that non-white patients were more affected than whites. The Lequesne questionnaire confirmed that women are more affected (P<0.05); 95.6% of the women were classified as having extremely severe or very severe cases. Furthermore, the Lequesne index showed that older patients were more severely affected (P<0.05).

Some comorbidities such as hypertension and diabetes were frequently present in patients in this study. The use of NSAIDs by a number of patients with OA may have elevated blood pressure.^14^ Furthermore, both hypertension and diabetes are described as having an impact in the pathophysiology of OA, and diabetes is currently considered a risk factor for progression of knee OA.^15^ Hypertension was the most frequent comorbidity, in 83.3% of the sample, followed by diabetes. Previous studies conducted in Brazil found a lower prevalence of hypertension, ranging from 59% to 81%, and diabetes ranging from 19% to 35%.^6^ ^,^ ^9^ ^,^ ^16^

Some international studies have described alcohol consumption as often relieving symptoms in patients with OA, but we are very familiar with the risks of this habit in the population in general.^17^ Moreover, recent studies have shown that drinking alcoholic beverages such as beer increases the risk of osteoarthritis.^18^ The current study found alcohol consumption in 20% of the sample. Perhaps the fact that most of the sample was female influenced this finding, since the female population is known to drink less alcohol than men. In order to confirm whether there was any relationship between drinking alcohol and the severity of OA, patients who consumed alcohol were correlated with Lequesne scores, but the outcome was not statistically significant (P>0.05).

Some years ago there were some doubts about the effects of cigarette smoking on patients with OA, but a recent meta-analysis showed that smoking did not have a protective effect.^19^ In our study, the prevalence of smokers was low (16.7%).

Recent studies show that bow-leggedness increases the incidence of OA and increases the progression of medial OA, so an increased prevalence of genu varum is expected in patients with OA of the knee.^20^ In the present study, more than 75% of the patients had the genu varum deformity.

The Lequesne questionnaire was developed in France in the 1970s and updated in 2003; it is used often in Europe and contains several questions for patients to answer about pain, discomfort and function, evaluating the severity of symptoms and degree of physical handicap.^21^ The Lequesne index, unlike other questionnaires, is quick and easy to apply, and non-subjective. It does not contain questions specific to the population of a given country and can therefore be used in any population. It is intended for patients with OA, and is brief, so responding is not difficult. Furthermore, it is difficult for patients to manipulate their score for their own benefit on this questionnaire, since they do not know which type of response has a higher point value. Of the total sample, 61.6% of cases were classified as “extremely severe” with a score >14. Of the total, 30% of the sample was considered “very severe,” 6.6% “severe,” and 1.6% “moderate.” The mean score was 15.53. The high score is consistent, because all these patients were referred for surgical treatment. Other patients with very high scores had rheumatoid arthritis as well as OA, and comprised 8.3% of the total. 

We also noted that although these patients were more severely affected according to their score, they faced a long wait time for surgery since few slots are available; slightly over 80% needed to wait 1-5 years for surgery.

## CONCLUSION

Most patients who seek medical help for this problem are over 65 years of age. Females were more prevalent, and whites were least prevalent. The most frequent comorbidity was hypertension. Lequesne index scores were higher in females and in the older patients in the sample, with statistically significant findings. The Lequesne scores for each patient were consistent with degree of severity recognized by the orthopedists and radiologists who recommended surgical treatment.
